# Low Back Pain Characteristics Among Health Science Undergraduates: A Prospective Study for 2-Year Follow Up

**DOI:** 10.3390/jcm15020684

**Published:** 2026-01-14

**Authors:** Janan Abbas, Saher Abu-Leil, Kamal Hamoud, Katherin Joubran

**Affiliations:** Department of Physical Therapy, Zefat Academic College, Zefat 13206, Israel; saher.a@zefat.ac.il (S.A.-L.); kamalh@zefat.ac.il (K.H.); katherin.jo@zefat.ac.il (K.J.)

**Keywords:** health science, students, low back pain, stress, females

## Abstract

**Background/Objectives:** Low back pain (LBP) is one of the most prevalent musculoskeletal disorders globally, significantly impacting quality of life across diverse populations. Despite its association with middle-aged and older populations, evidence indicates that LBP is increasingly prevalent among younger age groups. Health science students are considered a potential risk factor for LBP; however, longitudinal studies are scarce. This study aims to determine the risk factors for LBP among health science students over a 2-year follow-up. **Methods:** One hundred ninety-seven of the third-class health science students (Nursing, Physiotherapy, Medical laboratory science, and Emergency Medical services) were contacted in June 2024. A self-administered modified version of the Standardized Nordic Questionnaire, and data about sedentary and physical activity behavior, as well as 1-month LBP (lasting at least 12 h and numeric rating scale > 5) and stress scores, were recorded. **Results:** A total of 172/197 (87.3%) respondents completed the questionnaire at the end of the 2-year follow-up. The mean age was 25 ± 3.5 (years) and body mass index (BMI) value 23.5 ± 4.3 (kg/m^2^). About 49% (n = 84) and 20% (n = 34) of the participants had 1-month LBP and functional disability, respectively. No significant association was found between health science programs and the presence of 1-month LBP (*χ*^2^ = 0.55, *p* > 0.05). The logistic regression analyses found that males (OR = 0.269, *p* = 0.005) and a history of pain frequency (OR = 3.377, *p* = 0.001) had a significant association with LBP over time. **Conclusions:** This prospective study shows a high prevalence of 1-month LBP (48.8%) among health science students at Zefat Academic College. LBP was significantly related to sex (female) and pain frequency, but not to health science students. We believe that implementing ergonomic and educational strategies is recommended for this population.

## 1. Introduction

Low back pain (LBP) represents one of the most prevalent musculoskeletal disorders globally, constituting a leading cause of disability and significantly impacting quality of life across diverse populations [1]. According to the World Health Organization (WHO) LBP affected over 619 million people in 2020, with projections suggesting more than 843 million cases by 2050 if current trends continue [2]. While traditionally associated with middle-aged and older populations, evidence indicates that LBP is increasingly prevalent among younger age groups [3,4]. The WHO statistics show that the annual prevalence of LBP in young adults (20–44 years) is approximately 20–25%, much higher than in adolescents (10–19 years), and lower than middle aged (45–59 years). Additionally, some studies reported that a point prevalence (i.e., at the time of the study) of low back and musculoskeletal pain among university students ranges between 40–67% [5,6,7,8,9,10,11,12], and this prevalence could be greater in the health science discipline [11,13,14].

Among health science students, LBP has emerged as a particularly concerning public health issue, with implications extending beyond individual wellbeing to potentially affecting the future healthcare workforce. Health science students, including those pursuing degrees in nursing, physiotherapy, and related fields, demonstrate alarmingly high prevalence rates of LBP that often exceed those observed in the general population of similar age groups [7,15,16,17]. Additionally, it has been reported that medical students demonstrated a high prevalence of musculoskeletal pain compared to non-medical students [18]. This could be explained by the fact that health science undergraduates are at high risk for developing low back pain due to the demanding nature of their curriculum and lifestyle [10,19,20]. For example, nursing and physiotherapy students spend extended hours studying, attending lectures, and performing clinical practicing, which can result in prolonged sitting or standing postures, repetitive motions, and awkward body positions.

LBP is associated with multidimensional factors (e.g., physical and psychosocial) [21], with insufficient investigations among students in general and health science discipline in particular. Additionally, data regarding variables that are significantly associated with LBP are still ambiguous. Despite the growing recognition of LBP as a significant health concern among health science students, there is a notable absence of longitudinal studies that track the progression of LBP among students from their first class into the advanced years. A prospective study assesses changes in low back pain over time, distinguishing new, persistent, and resolved cases rather than providing a single prevalence estimate. By establishing temporal sequencing between baseline factors and later outcomes, it strengthens inference about potential predictors and offers greater clinical relevance for prevention and early intervention. Based on previous studies, we hypothesized that being a health science student with a unique lifestyle increases the risk for LBP onset.

Thus, the aims of this study are (1) to establish whether health science programs are associated with LBP and (2) to define the factors that associated with the onset of LBP among health science undergraduates through a longitudinal study.

## 2. Materials and Methods

### 2.1. Study Design

This prospective study among health science undergraduates (full-time Bachelor’s degree), aged ≥18 years, including nursing, physical Therapy, medical laboratory sciences, and emergency medical services, was conducted during a 2-year follow-up period.

### 2.2. Sample and Instruments

This study was started in June 2022 and included a baseline of 197 students, who were in the 2nd semester of their first year at Zefat Academic College located in the north of Israel. Data collection was completed in June 2024, with 172 students responding at the follow-up. Subjects were followed up once in the 2nd semester of the third year of the academic program, and all have completed at least half of their clinical practices. Participants were invited and contacted in their classrooms by one of the study’s assessors (without acquaintance or relationship with students) at the end of the course in the presence of the teacher. Participation was voluntary and anonymous, and subjects were assigned a special code number, with no personal identifying information accessible to the authors, thereby minimizing potential academic conflicts of interest. A consent form, including the study’s purpose and the student’s right to withdraw at any time, was provided to each participant. The Department of Research Ethics Committee at Zefat Academic College approved this study (no. 19-2022).

We used the same instruments and measures following the baseline recruitment [22]. These measures included (a) a structured and anonymous modified questionnaire of the Nordic questionnaire [23], which seeks information on sociodemographic characteristics and smoking habits as well as data about sedentary lifestyle and physical activity [24,25]. One-year LBP was defined as any self-reported episode of pain occurring between the end of the second semester of the second academic year and one month prior to follow-up data collection (June 2023–May 2024). The 1-year LBP assessment included pain frequency, disability, seeking care, and medication use. Pain frequency was measured on an ordinal scale ranging from high frequency (daily) to low frequency (rarely or none). LBP-related disability, seeking care, and medication use were assessed as dichotomous (yes/no) variables. A composite 1-year LBP score was then constructed based on four variables (e.g., pain frequency and disability), each dichotomized as 0 (absent) or 1 (present). The total score was calculated by summing the coded items, yielding a range from 0 to 4, with higher scores indicating greater LBP burden.

Functional disability associated with LBP was assessed using the Oswestry Disability Index (ODI) [26] and referred to the month preceding data collection (May to June 2024). Stress-related variables including educational, family, personal, and social stressors were evaluated over the same period [23]. Each stress domain was initially assessed using a four-level response scale (very high, high, little, or none). For analytical purposes, responses were dichotomized into absence (0) or presence (1) of stress, and a composite stress score was calculated by summing the four binary indicators, resulting in a total score ranging from 0 to 4. Higher scores reflected greater cumulative stress exposure. One-month low back pain was defined as LBP occurring within the month prior to data collection, with a minimum duration of 12 h and a numeric rating scale score exceeding 5 [27].

### 2.3. Statistical Analysis

A post hoc power analysis, based on the final sample size (n = 172) and the observed 1-month LBP prevalence (48.8%), indicated that, at a two-sided α level of 0.05, the study had approximately 80% statistical power to detect moderate effect sizes, corresponding to absolute prevalence differences of approximately 15–17% or greater. However, the study was underpowered to detect small effect sizes, particularly in subgroup analyses, which may have increased the risk of type II error.

We used statistical analysis methods with IBM SPSS version 25. We first checked whether the numerical data followed a normal distribution. To evaluate changes in 1-month LBP prevalence, McNemar’s test was conducted: new cases of LBP refer to participants reporting LBP at the follow-up but not at baseline; whereas recovered-LBP refers to participants reporting LBP at the baseline but not at the follow-up. Paired T-tests, as well as two one-way ANOVAs and logistic regression (Method-Enter, dependent variable-1-month LBP, independent variable- age, sex, sedentary behavior, etc.) analysis were also used. In the regression model, we included all variables that have the potential to be related to LBP according to previous studies. A significant reference was set at *p* < 0.05.

## 3. Results

### 3.1. Demographic Features

**Figure 1:**
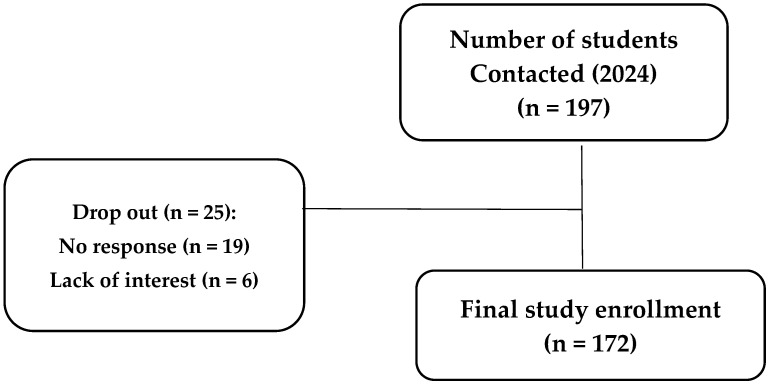
Flow chart for the study sample after 2-year follow up.

One hundred seventy-two out of 197 (87.3%), responded and completed the questionnaire at the end of the 2-year follow-up (Figure 1). Seventy-six percent of participants were females (n = 131), and 41.3% (n = 71) were from the nursing program. The mean age and body mass index (BMI) of the participants were 25 ± 3.5 (years) and 23.5 ± 4.3 (kg/m^2^), respectively (Table 1). In addition, 58.7% of the study sample were involved in physical activity. Approximately 80% of students engaged in prolonged sitting (up to 5 h), and they reported study-related stress ranging from very high to quite high.

Our findings show that health science students in general have a significantly lower stress score over time, yet with a small effect: (Post) 3.10 ± 1.10 vs. (Pre) 3.33 ± 0.87, *t*(171) = −2.42, *p* = 0.016; Cohen’s *d* = 0.19. Notably, no significant difference in stress score was observed between academic departments *(F*_(3, 168)_ = 1.34, *p* > 0.05, *R*^2^ = 0.006).

### 3.2. Low Back Pain Characteristics

The results indicate that 48.8% (n = 84) and 19.8% (n = 34) of the cases had 1-month LBP and functional disability, respectively. The features of 1-year LBP at the follow-up were 70.3% (n = 121 out of 172) with LBP, frequencies varied from every day to once a month, 27.9% (n = 48) decreased their daily activity (disability), 22.1% (n = 38) sought care, and 25% (n = 43) used medication (Table 2). Notably, the percentage of females reporting LBP increased over time (55.7% vs. 47.3%).

Despite a slight increase in 1-month LBP reported over time, no significant difference was found (χ^2^_(1, 172)_ *=* 0.55, *p* > 0.05). For example, 36 participants (20.9%) reported new-onset LBP, compared with 29 (16.9%) who had recovered LBP at follow-up. Similarly, no significant difference was observed in the 1-year LBP score among all students between baseline and follow-up (1.45 vs. 1.31, *p* = 0.188) (Table 3).

**Figure 2:**
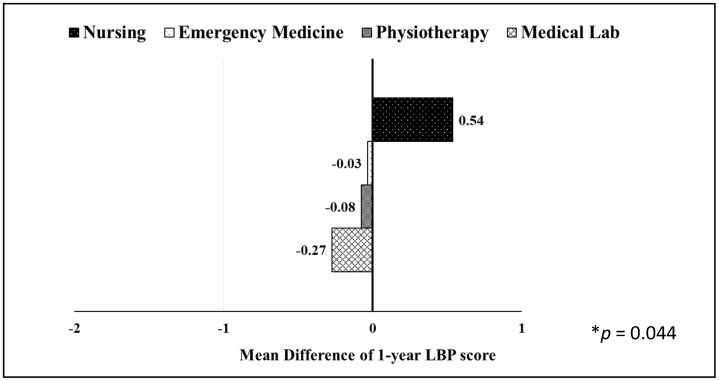
Depicts changes in 1-year LBP scores by department (baseline vs. follow-up). Negative values depict improvement in LBP. * *p* value represents only the difference between nursing and medical lab (adjustment for multiple comparisons: Sidak).

Department-specific analyses yielded comparable findings, with the exception of the nursing program, which demonstrated a significant increase in the 1-year LBP score over time (1.15 vs. 1.69, *p* = 0.002; Table 3). Notably, comparisons across departments revealed a statistically significant overall difference in the mean change in 1-year LBP scores (*F*_(3, 168)_ = 3.33, *p* = 0.024), although the effect size was small (R^2^ = 0.038; partial η^2^ = 0.055; Figure 2). Specifically, students in the medical laboratory program exhibited the greatest improvement in LBP scores (M = −0.27, SD = 1.55), followed by physiotherapy (M = −0.08, SD = 1.29) and emergency medical services (M = −0.03, SD = 1.52), whereas nursing students demonstrated a worsening trend (M = 0.54, SD = 1.37).

The logistic regression analyses (method-enter, dependent variable-1-month LBP) following the baseline data, noted that both males (OR= 0.269, *p* = 0.005) and a history of LBP frequency at the first year (OR= 3.377, *p* = 0.001) were significantly associated with LBP at the 2-year follow-up (Table 4). Neither physical activity nor sitting behavior was associated with LBP.

## 4. Discussion

This 2-year prospective study design with a relatively high follow-up rate (87%), which shed light on the temporal patterns of LBP among future health professionals, investigated the prevalence and predictors of low back pain (LBP) among health science undergraduates in Zefat Academic College.

The findings revealed a high prevalence of both 1-month (48.8%) and 1-year (70.3%) LBP, aligning with previous studies [22,28] as well as reports obtained from health science undergraduates worldwide [29,30,31,32,33]. These rates are substantially higher than those reported in the general young adult population [2], emphasizing the vulnerability of students in physically and mentally demanding programs such as nursing and physiotherapy. In addition, the small decrease in stress score observed over time (*p* = 0.016; Cohen’s *d* = 0.19) should be interpreted with caution, as the study was underpowered to reliably detect small effect sizes.

The findings showed that being female and a higher frequency of pain history were associated with an increased likelihood of reporting LBP over time. These results suggest that students who experienced more frequent pain during their first academic year were more likely to report LBP at follow-up. The association between a history of LBP and the onset of pain has previously been reported [22,34,35,36]. To the best of our knowledge, there have been limited longitudinal studies conducted among students. For example, a prospective cohort study (4 years) among nursing students has reported that a history of LBP was considered a predictive factor for new episodes [36]. Marchado and colleagues have shown that one-third of patients in the general population will experience a recurrent episode of pain after an acute episode of LBP [34]. The authors noted that experiencing more than 2 previous episodes of LBP triples the odds of recurrence within 1 year. A recent cross-sectional study using machine-learning analysis also found that a history of pain frequency had the greatest impact on LBP among health science students [22].

The association between females and LBP is in agreement with many studies among students and general populations [17,30,37,38,39,40] but contradicts others [31,41,42]. A recent global burden of LBP from 1990 to 2021 has reported that females have higher prevalence rates of LBP than males across all ages [43]. Alanazi and Kashoo have recently reported that female students demonstrated higher musculoskeletal pain than males [44]. A review study has also noted that women are affected more frequently by LBP than men [45]. We hypotheses that this outcome could be related to the biological differences, psychosocial and metabolic aspects, as well as genetics and hormonal factors that contribute to the observed sex differences and act as sex-specific pain mediators [46,47,48].

The logistic regression model’s overall explanatory power was modest, as reflected by the Nagelkerke R^2^ value (0.215). This suggests that a substantial proportion of the variance in low back pain outcomes remains unexplained by the included predictors. While statistically significant associations were identified (e.g., sex), the model suggests that low back pain in this population is influenced by additional unmeasured factors. Notably, biopsychosocial, ergonomic, and physical variables such as sleep quality, academic workload, clinical exposure intensity, muscle endurance, and objective postural or biomechanical measures were not incorporated and may have contributed meaningfully to outcome variability.

No significant association was found between health science studies and the presence of 1-month LBP over 2 years of follow-up (*χ*^2^ = 0.55, *p* > 0.05). The finding showed that 20.9% (n = 36) of participants reported a new onset of LBP, whereas about 17% (n = 29) had recovered from pain compared to the baseline. Additionally, no significant differences were noted in the 1-year LBP score outcomes, except for the nursing department, which presented worsening over time. Our findings could partially challenge the notion that health science studies increase the risk for LBP [10,29,49,50]. For example, Falavigna and colleagues have clearly demonstrated the association between physiotherapy undergraduates’ study and LBP [10]. A review and meta-analyses study among nurses and medical students declared from strong and moderate evidence that students of the final year of study were associated with a higher 12-month low back pain prevalence in both student groups [49]. On the other hand, a literature review and prospective cohort study among undergraduate nurses declared that there was no significant change on LBP prevalence over time [51].

The plausible explanation of the lack of relationship between health science students and 1-month LBP in the current study could be mainly attributed to (1) a decrease in 1-month stress score, (2) the fact that half of the participants are physically active, and (3) students, particularly in physiotherapy, receive some back school education during the academic program. The absence of an a priori statistical power analysis, despite the overall follow-up rate being high, could consequently be insufficient to detect, particularly within departmental subgroups, an association between health science programs and low back pain outcomes. This may also partly explain the divergent trends observed in the 1-year LBP score between the nursing program and the other departments.

Although factors related to LBP have been extensively investigated in the literature, to our knowledge, few prospective studies have been conducted in both general and student populations. A 1-year prospective study by Kanchanomai and colleagues (2015) among healthy Thai students previously reported that a lack of low-back support during computer work and quadriceps muscle tightness are significantly associated with LBP [52]. Another prospective study (2010) among female nursing students has demonstrated that smoking, increased physical activity, higher stress, greater pelvic rotation, and reduced muscle endurance were significant and independent predictors for new onset of LBP [36]. Two prospective studies among British schoolchildren (11–14 years) and newly female health care workers have previously reported that emotional problems and somatic symptoms, as well as high physical work load, were significantly associated with LBP, respectively [53,54]. The diversity of the studies’ outcomes regarding the factors related to LBP is primarily due to the multi-dimensional nature of this phenomenon; therefore, researchers should consider all these variables. Additionally, reflecting the potential variations of the studies, such as the sample (e.g., students, schoolchildren), methodology, and design (e.g., cross-sectional or prospective study), is required.

### 4.1. Clinical Implication

Although no significant association has been found between health science studies and LBP over time, educational institutions should therefore prioritize early, female-related LBP prevention and management within health science curricula. The fact that a history of pain is associated with reporting LBP at follow-up suggests that implementing ergonomic strategies, such as ensuring adequate lumbar support and scheduled movement breaks during long lectures and prolonged sitting, is recommended.

### 4.2. Limitations of the Study

This study has several limitations. It was conducted at a single academic institution, which may limit generalizability to other health science programs or educational settings. The outcomes were based on self-reported questionnaires, introducing potential recall and reporting bias, particularly for the 1-year LBP measures. Additionally, pain assessment focused primarily on prevalence, duration, and disability, without detailed evaluation of pain characteristics or quality (e.g., pain type, radiation, or neuropathic features), limiting insight into the clinical heterogeneity of LBP. The absence of objective clinical, biomechanical, or ergonomic assessments prevents validation of reported symptoms and identification of underlying physical contributors. Additionally, data were collected at only two time points, restricting the ability to capture changes and patterns of symptoms over time. Finally, the lack of an a priori power analysis, together with the limited explanatory performance of the regression model, constrained the interpretability of the results and prevented causal conclusions. Relatively small sample sizes within subgroups and the absence of relevant covariates including sleep quality, clinical workload, and psychosocial stressors may have introduced residual confounding, underscoring the need for cautious interpretation of the findings.

## 5. Conclusions

The results showed a high prevalence of 1-month LBP (48.8%) among health science students. Being a health science undergraduate was not associated with LBP. Females and a history of pain are significantly associated with the presence of LBP at follow-up. Based on the preliminary observations of the present study, the implementation of educational and ergonomic strategies may be considered, although these recommendations are not based on interventional evidence. We also believe that future longitudinal research should incorporate objective measures and a broader set of validated predictors to improve the accuracy and clinical relevance of LBP risk models.

## Figures and Tables

**Table 1:** Sample size characteristics at 2-year follow up.

Variable	n (%)/or Mean ± SD
Male	41 (23.8)
Female	131 (76.2)
Mean age (year)	25.1 ± 3.5
Mean BMI (kg/m^2^)	23.5 ± 4.3
Physical activity (yes)	101 (58.7)
Ethnicity:	
Jews	64 (37.2)
Arabs	108 (62.8)
Prolonged daily sitting:	
up to 5 h	139 (80.8)
>5 h	33 (19.2)
* Study-related stress:	
Very high–quite high	134 (77.9)
Little–none	38 (22.1)
Department:	
Nursing	71 (41.3)
Physical therapy	39 (22.7)
Medical Lab	33 (19.2)
Emergency Medical Services	29 (16.9)

BMI—Body mass index, SD—standard deviation. * Stress-related variable appeared alone, as this variable was the most common compared to other types of stress.

**Table 2:** Low back pain characteristics of the study sample at 2-year follow up and baseline.

	Follow-Up n (%)	Baseline n (%)
“1-year LBP”	121 (70.3)	106 (61.6)
1. Frequency of LBP:		
Every day	40 (23.3)	29 (16.9)
Once a week	46 (26.7)	39 (22.7)
Once a month	35 (20.3)	38 (22.1)
Rarely/never	51 (29.7)	66 (38.4)
2. Disability	48 (27.9)	54 (31.4)
3. Seeking care	38 (22.1)	30 (17.4)
4. Medication consumption	43 (25)	35 (20.3)
1-month LBP:	84 (48.8)	77 (44.8)
Males	11 (26.8)	15 (36.5)
Females	73 (55.7)	62 (47.3)
1-month functional disability	34 (19.8)	NA

NA—not applicable.

**Table 3:** Paired *t*-test for “1-year LBP” score over time for the study sample, by department.

1-Year LBP Score	Period	Mean	SD	T	*p* Value	Cohen’s d
All participants	Pre Post	1.31 1.45	1.276 1.281	−1.332	0.188	0.109
Medical lab	Pre Post	1.48 1.21	1.325 1.219	1.013	0.319	0.215
Physio.	Pre Post	1.28 1.21	1.234 1.196	0.374	0.711	0.059
Emergency med.	Pre Post	1.52 1.48	1.502 1.271	0.122	0.904	0.028
Nursing	Pre Post	1.15 1.69	1.178 1.337	−3.288	**0.002**	0.425

SD—standard deviation.

**Table 4:** Variables that were associated with 1-month LBP over the years following the logistic regression.

Variable	OR	95% CI	*p* Value
Department			0.994
Nursing (Reference)	-	-	-
Medical Lab	1.140	0.454–2.863	0.780
Physiotherapy	1.028	0.364–2.900	0.959
Emergency Medicine	1.043	0.378–2.872	0.936
Age	0.958	0.851–1.079	0.482
Ethnicity (Jews)	1.080	0.494–2.385	0.846
Sex (Male)	0.269	0.107–0.676	**0.005**
BMI	1.019	0.931–1.116	0.677
Smoking pre (Yes)	1.064	0.393–2.879	0.903
Physical activity pre (Yes)	0.909	0.448–1.846	0.792
Prolonged daily sitting (pre)	1.203	0.819–1.768	0.345
Seeking care pre (yes)	0.683	0.271–1.724	0.420
Frequency of LBP pre (yes)	3.377	1.605–7.105	**0.001**
Study-related stress pre (yes)	1.725	0.807–3.689	0.160

OR—odds ratio, CI—confidence Interval, Nagelkerke R^2^ = 0.215.

## Data Availability

The data presented in this study are available upon request from the corresponding author due to privacy reasons.
